# Charity Commission's Memorandum

**Published:** 1916-09-16

**Authors:** 


### THE WAR CHARITIES ACT, 1916.

### Charity Commission's Memorandum.

The Charity Commission have prepared the following Memorandum, dated August 1916, for the use of the public. We have inserted cross-heads for the convenience of our readers :

War Charities Must be Registered.

1. The Act provides in Section 1 (1) that it shall not be lawful to make a public appeal for money or articles in kind for any war charity as defined by the Act or to attempt to raise money by a bazaar, sale, entertainment, or exhibition, or by any similar means for any such charity unless (1) the charity is registered under the Act and (2) the charity has approved of such appeal or attempt to raise money. Any contravention of this provision is an offence against the Act, and is punishable on summary conviction by a fine of not more than £100 or imprisonment with or without hard labour for not more than three months.

Definition of a War Charity.

2. The expression "war charity" is defined to mean any fund, institution, or association having for its object or amongst its objects the relief of suffering or distress, the supply of needs or comforts, or any other charitable purposes connected with the present war, unless in the case of a charity established before the commencement of the war such object is subsidiary only to the principal purposes of the charity. The expression does not include the Royal Patriotic Fund Corporation or the Statutory Committee or any local or district committee established under the Naval and Military War Pensions, etc., Act, 1915. Any question whether a charity is a war charity is to be finally determined by the Charity Commissioners. Application for determination of the question whether a charity is or is not a war charity must be made to the Charity Commissioners in a form, copies of which can be obtained from the Office of the Commission, Ryder Street, St. James's, London, S.W.

Collections Outside the Act.

3. The Act does not apply

(1) to any collection at Divine service in a place of public worship, or

(2) to any charity which may be exempted by the registration authority from the provisions of the section; and an appeal or attempt to raise money for an unregistered charity will not be an offence against the Act if made with the approval of the committee or governing body of a charity before the expiration of a period of one month from the passing of the Act (August 23, 1916) or pending the decision of the authority on an application for registration made within such month [Section 1 (2)]. No proceedings for an offence against the Act may be taken except by or with the approval of the Charity Commissioners.

Registration Authorities.

4. The registration authority is :

(*a*) As respects the City of London—the Common Council of the City,

(*b*) As respects a Municipal Borough or Urban District—the Council of the Borough or Urban District,

(*c*) Elsewhere—the County Council.

Conditions of Registration.

5. The conditions required to be observed in order to entitle a charity to registration are shortly

(*a*) That it shall be administered by a responsible committee or other body of not less than three persons,

(*b*) That proper minutes shall be kept,

(*c*) That proper books of accounts shall be kept and regularly audited by some person approved by the authority and that copies of such accounts shall be sent to the authority,

(*d*) That all monies received shall be paid into a separate account at a bank,

(*e*) That such particulars with regard to accounts and other records shall be furnished as may be required by the authority or by the Commissioners,

(*f*) That the books and accounts shall be open to inspection by any person authorised by the authority or by the Commissioners.

Registration or Exemption.

6. Application for registration or exemption must be made to the authority in whose area the administrative centre of the charity is situate and must be made in the forms prescribed by the Charity Commissioners, copies of which forms can be obtained from the authority. Upon registration or exemption of a charity a certificate of registration or exemption will be given to the charity by the authority.

Regulations for Exemption.

7. The regulations made by the Charity Commissioners and approved by the Secretary of State provide that no charity may be exempted from the provisions of Section 1 of the Act unless the scope of its operations as regards the amount of subscriptions expected to be received, the duration of the charity or the area of collection or benefit is so limited as in the opinion of the authority to make it unnecessary in the interests of the public that the charity should be registered under the Act.

Appeals.

8. An appeal against a refusal to register a charity will lie to the Charity Commissioners.

Copies of the Act, 1½d. each, and of the regulations, 1d. each, can be obtained from Messrs. Wyman and Sons, Ltd., Fetter Lane, London, E.C.

